# A decade later: a longitudinal retrospective cohort study of the clinical course of childhood-onset epilepsy in a multi-ethnic tertiary centre

**DOI:** 10.1186/s42494-026-00277-z

**Published:** 2026-09-04

**Authors:** Jen Ern Tan, Juen Kiem Tan, Michelle Maryanne Tan, Zhi Xuan Ng, Zhee Yee Chan, Zi Qi Ang, Nur Arifah Mohd Khaili, Nur Liyana Rahmat, Jasmine Lee, Ching Soong Khoo, Hui Jan Tan, Sau Wei Wong

**Affiliations:** 1 Neurology Unit, Paediatric Department, Hospital Tunku Ampuan Besar Tuanku Aishah Rohani, Universiti Kebangsaan Malaysia, Kuala Lumpur, 56000 Malaysia; 2 Department of Medicine, Faculty of Medicine, Universiti Kebangsaan Malaysia, Kuala Lumpur, 56000 Malaysia; 3 Neurology Unit, Department of Medicine, Hospital Canselor Tuanku Muhriz, Kuala Lumpur, 56000 Malaysia; 4 Department of Medicine, Hospital Tengku Ampuan Rahimah, Selangor, 41200 Malaysia; 5 Paediatric Department, Faculty of Medicine, Hospital Tunku Ampuan Besar Tuanku Aishah Rohani, Universiti Kebangsaan Malaysia, Kuala Lumpur, 56000 Malaysia

**Keywords:** Seizure, Childhood, Epilepsy, Neurodevelopmental outcomes, Transition of care

## Abstract

**Background:**

Epilepsy affects a significant proportion of people globally, with many cases beginning in childhood. However, there is a lack of longitudinal studies exploring the long-term outcomes and characteristics of childhood-onset epilepsy into adulthood. This study aimed to analyze the trajectory of childhood-onset epilepsy to identify particular characteristics and outcomes that could help guide future management.

**Methods:**

This retrospective cohort study was conducted between June 2021 and December 2022 at adult and paediatric clinics. Participant data were obtained from medical records and corroborated through history taking during follow-up visits.

**Results:**

A total of 100 participants diagnosed with epilepsy since childhood were included. The mean age of the cohort was 23.15 (SD = 8.80) years, with a mean age at epilepsy diagnosis of 3.97 years. Focal seizures were the most prevalent type (53%), with structural causes being the predominant aetiology of epilepsy. A total of 48% of participants were identified with intellectual disabilities, 24% with learning disabilities, and 32% with behavioural difficulties. Consequently, 45% required enrollment in special education institutions. We did not observe a statistically significant difference in seizure control over the 10-year follow-up period, and no analyzed factors were significantly associated with seizure control.

**Conclusions:**

Childhood-onset epilepsy is associated with substantial long-term neurodevelopmental and psychosocial consequences. Our findings highlight the need for individualized, multidisciplinary management and clear, structured transition pathways from paediatric to adult neurology services. We outline several recommendations to enhance overall management as patients transition into adulthood.

## Background

Epilepsy is defined as a brain disorder characterized by an enduring predisposition to generate epileptic seizures [1]. Based on a systematic review and meta-analysis of international studies published in 2017, the global prevalence of epilepsy is 7.6 per 1000 population, with a higher prevalence observed in childhood-onset epilepsy [2]. In Malaysia, the prevalence is 7.8 per 1000 population, comparable with global statistics [3].

International studies suggest that up to two-thirds of people with epilepsy (PWE) eventually achieve terminal remission and disease resolution after several years of treatment [4–6]. Seizure freedom was defined as the absence of seizure episodes for at least 12 months or the duration equal to 3 times the longest seizure-free interval before intervention, whichever was longer. Treatment failure is when the seizure recurs despite adequate intervention. Patients who remained seizure-free for three times the preintervention interseizure interval but for less than 12 months were classified as having an undetermined outcome [7]. 

Various studies have shown that inaccurate diagnosis and suboptimal management with different treatment protocols contribute to uncontrolled epilepsy [8], which is associated with cognitive impairment, psychosocial dysfunction, and increased risk of sudden unexpected death in epilepsy (SUDEP) [9]. Due to the heterogeneity of epilepsy aetiologies and outcomes, identifying factors influencing seizure control and optimizing treatment is critical for prognosis [10, 11]. Studies are essential to provide a better understanding of the life cycle of epilepsy and to evaluate treatment effectiveness [12]. At present, studies examining the course and outcomes of childhood-onset epilepsies are lacking.

In this study, we retrospectively analysed a cohort of PWE with at least ten years of follow-up to examine the long-term clinical trajectories of childhood-onset epilepsy and identify factors influencing seizure control.

## Methods

### Study design

This retrospective cohort study was conducted at the adult and paediatric epilepsy clinics of Hospital Canselor Tuanku Muhriz, Universiti Kebangsaan Malaysia, from June 2021 to December 2022. Ethical approval was obtained from the local Ethics and Research Board (Universiti Kebangsaan Malaysia Research and Ethics Committee; Research code: JEP-2021-594). All PWE with epilepsy onset before age 12 and at least 10 years of follow-up were recruited via universal sampling. PWE were diagnosed based on the International League Against Epilepsy (ILAE) criteria. We excluded those who presented with febrile or provoked seizures with an aetiology such as encephalitis, toxins, severe electrolyte imbalances, and the presence of space-occupying lesions, such as brain tumours or abscesses.

Participant data were manually extracted from the medical records department and entered into a data collection sheet. Demographic data collected included current age, age of onset, activities of daily living, and significant family history or developmental history. Clinical data, such as intellectual and behavioural issues, seizure types, aetiology, electroencephalography (EEG) reports, brain imaging, medications, and long-term impact on education, were documented. Additional information was corroborated with participants during clinical visits.

### Sample size

Sample size calculation was based on our annual follow-up census at the adult and paediatric epilepsy clinics. On average, 420 patients are reviewed each year, with an estimated 10% of the patients meeting the inclusion criteria. A 95% confidence level and 0.05 margin of error were used, with *Z* set at 1.96.

We used the following sample size formula for a finite population:

Infinite sample size: $n = [{Z^2}p (1-p)]/{C^2}$  

Finite sample size: $\acute{n} = n/[1 + (n-1)/Pop]$  

*Z* = 1.96

*p* = 0.1 (percentage of population)

*C* = 0.05 (confidence level)

*Pop* = 420 (population)

Therefore,

Infinite sample size: $n = [{Z^2}p (1-p)]/{C^2}$  

$$n = (1.96)^2\times0.1\times(1-0.1)/(0.05)^2$$  

*n* = 138.3

Finite sample size: $\acute{n} = n/[1 + (n-1)/Pop]$

$$\acute{n}= 138.3/[1 + (138.3-1)/420]$$  

*ń* = 105

Thus, 105 patients were required to achieve 95% confidence with a 5% margin of error.

### Statistical analysis

All data were encoded and analysed using the Statistical Package for the Social Sciences (SPSS) Version 26. A 95% confidence interval was reported where applicable, with significance set at a *P*-value < 0.05. Categorical variables were reported as frequencies and percentages, while continuous variables with a normal distribution, such as age and age at diagnosis, were reported as mean ± standard deviation (SD). Chi-square test was used to compare qualitative variables and seizure control over 10 years. The Wilcoxon signed-rank test evaluated differences in seizure control from baseline up to 10-year follow-up. Furthermore, a logistic regression analysis was performed to identify predictors of seizure control.

## Results

### Demographic and clinical profile

After screening 117 patients, we included 100 participants diagnosed with epilepsy before age 12. The mean age of the participants was 23.15 (SD = 8.80), ranging from 11 to 46 years (Table 1). Epilepsy was diagnosed at a mean age of 3.97 years, with the youngest diagnosed at the age of 10 months and the oldest at 12 years, with an equal distribution of both sexes. Focal seizures were the most prevalent (53%), followed by generalized epilepsy (31%). Structural causes were the predominant cause of epilepsy, while 33% and 13% of the aetiologies were unknown and genetic, respectively.

A large proportion (48%) of participants had an intellectual disability, 24% had a learning disability, and 28% were intellectually normal. Most participants (68%) had no behavioural issues. Among those affected, autism spectrum disorder (ASD, 10%) and attention deficit hyperactivity disorder (ADHD, 6%) were the most common. Over three-quarters (76%) of the subjects were independently mobile, while 17% required assistance with activities of daily living (ADL). Only a minority (12%) had a family history of epilepsy, 16% had a history of febrile seizures, and 12% had perinatal asphyxia.

Interictal EEG showed focal epileptiform discharges in 68%, generalized discharges in 12%, and normal findings in 11%. Ictal EEG was unavailable for more than half of the patients. However, among those with ictal EEG, focal epileptiform discharges predominated (28%). The most common imaging findings were malformation of cortical development (MCD, 30%) and hypoxic-ischemic encephalopathy (HIE, 11%).

Seizure control over 10 years was also reviewed. Notably, participants who were not seizure-free from the initial onset of epilepsy had a predisposition to develop further seizure episodes. Comparison of baseline and post-treatment EEG profiles revealed that focal epileptiform discharges decreased from 68 cases at baseline to 35 post-treatment, yielding a 48.5% normalization rate. In comparison, generalized epileptiform discharges decreased from 12 cases at baseline to 9 post-treatment, representing a 25.0% normalization rate.

**Table 1:** Demographic and clinical profile of patients

Demographic characteristic	Frequency, *n* (%)	*P*-value (onset)	*P*-value (10 years)
Age (years)*		0.367	0.138
Mean ± standard deviation	23.15 (SD = 8.80)		
Range	11 – 46		
**Age Group**		0.361	0.436
7 to 17	35 (35.0)		
18 to 25	29 (29.0)		
More than 25	36 (36.0)		
**Age at diagnosis (years)**		0.079	0.373
Mean ± standard deviation	3.97 (SD = 3.56)		
Range	0.10 – 12.00		
**Sex**		0.370	0.500
Male	50 (50.0)		
Female	50 (50.0)		
**Seizure type**		0.093	0.250
Focal	53 (53.0)		
Generalised	31 (31.0)		
Both	16 (16.0)		
**Seizure aetiology**		**0.006**	0.079
Structural	50 (50.0)		
Genetic	13 (13.0)		
Structural and genetic	2 (2.0)		
Infection	1 (1.0)		
Immune	1 (1.0)		
Unknown	33 (33.0)		
**Subject intellectual status**		0.421	0.199
With learning disability	24 (24.0)		
With intellectual disability	48 (48.0)		
Normal	28 (28.0)		
**Subject behavioural issues**		**0.004**	0.289
None	68 (68.0)		
Autism spectrum disorder (ASD)	10 (10.0)		
Attention deficit hyperactivity disorder (ADHD)	6 (6.0)		
Major depressive disorder (MDD)	4 (4.0)		
Mixed ADHD-ASD	3 (3.0)		
Aggression	2 (2.0)		
Anxiety	1 (1.0)		
Psychosis	1 (1.0)		
Adjustment disorder	1 (1.0)		
Conversion disorder	1 (1.0)		
Immature behavior	1 (1.0)		
Obsessive-compulsive disorder (OCD)	1 (1.0)		
Suicidal attempt	1 (1.0)		
**Subject's mobility**		0.227	0.446
Independent	76 (76.0)		
With support	13 (13.0)		
Unable to ambulate	11 (11.0)		
**Subject's performance of activities of daily living (ADL)**		0.294	0.422
Independent	60 (60.0)		
Partly independent	17 (17.0)		
Dependent	23 (23.0)		
**Family history of epilepsy**		0.310	0.112
No	85 (85.0)		
Yes	12 (12.0)		
Unknown	3 (3.0)		
**History of febrile seizures**		0.179	0.343
No	84 (84.0)		
Yes	16 (16.0)		
**Evidence of perinatal asphyxia**		**0.032**	0.500
No	88 (88.0)		
Yes	12 (12.0)		
**Inter-ictal EEG results**		0.430	0.466
Focal epileptiform discharges	68 (68.0)		
Generalised epileptiform discharges	12 (12.0)		
No abnormality detected	11 (11.0)		
Not available	9 (9.0)		
**Ictal event EEG**		0.226	0.269
Focal epileptiform discharges	28 (28.0)		
Generalised epileptiform discharges	7 (7.0)		
No recorded event	4 (4.0)		
Not available	61 (61.0)		
**CT-MRI results**		0.438	0.390
Malformation of cortical development (MCD)	30 (30.0)		
Hypoxic-ischemic encephalopathy (HIE) changes	11 (11.0)		
Insignificant change	5 (5.0)		
Hippocampal sclerosis	5 (5.0)		
Tumours	4 (4.0)		
Traumatic brain injury	1 (1.0)		
No abnormality detected	30 (30.0)		
Not available	14 (14.0)		
**Seizure characteristic at 10 years**		**Frequency, ** ***n*** ** (%)**	
Focal		53 (53.0)	
Generalised		35 (35.0)	
Focal and generalised		12 (12.0)	

*Age listed represents the participant’s age at recruitment

Over one-third of participants received dual ASM therapy (36%), followed by polytherapy (34%) and monotherapy (29%) (Table 2). The most commonly prescribed ASMs were carbamazepine (CBZ, 49%) and sodium valproate (VPA, 49%). The median number of medications prescribed was 2 (IQR 2), ranging from 1 to 7. Most patients were compliant with treatment (82%) and did not report adverse effects (76%). Among those who experienced adverse effects, drowsiness, dizziness, and weight gain were the most frequently reported (3% each). Overall, 8% of patients received non-pharmacological treatments, including ketogenic diet therapy (4%), epilepsy surgery (3%), and neuromodulation (1%).

After initiation of ASMs, EEG showed no abnormalities in more than half of patients (56%), followed by focal epileptiform discharges (35%) and generalized epileptiform discharges (9%).

**Table 2:** Treatment characteristics and post-treatment EEG findings

Treatment characteristics	Frequency (%)	*P-value (Onset)	*P-value (10 years)
**Antiseizure medications, IQR **(25^th^, 75^th^ Percentile)			
Monotherapy	29 (29.0)	0.011	0.006
Dual therapy	36 (36.0)		
Polytherapy	34 (34.0)		
No therapy	1 (1.0)		
Specific antiseizure medications*			
Carbamazepine	49 (22.2)	0.091	0.39
Sodium valproate	49 (22.2)		
Levetiracetam	37 (16.7)		
Topiramate	23 (10.5)		
Clobazam	20 (9.0)		
Clonazepam	17 (7.8)		
Lamotrigine	9 (4.2)		
Acetazolamide	5 (2.3)		
Others	11 (5.0)		
None	1 (0.1)		
**Compliance to treatment**			
Yes	82 (82.0)	0.101	0.344
No	18 (18.0)		
**Non-pharmacological treatment**			
Ketogenic diet	4 (4.0)	0.405	0.378
Epilepsy surgery (lesionectomy, corpus callostomy, hemispherectomy)	3 (3.0)		
Neuromodulation (VNS, DBS, RNS)	1 (1.0)		
None	92 (92.0)		
**EEG results after treatment initiation**			
Focal epileptiform discharges	35 (35.0)	0.399	0.484
Generalised epileptiform discharges	9 (9.0)		
No abnormality detected	56 (56.0)		

IQR= Interquartile range; VNS= vagus nerve stimulation; DBS= Deep brain stimulation; RNS=Responsive neurostimulation

*chi-square

### Long-term social impacts of seizures

Seizures can have significant long-term consequences, particularly in terms of educational attainment and disability status. In this cohort, 45% of participants attended special schools, while 14% received no formal education (Table 3). Among the 41% who attended standard schools, 7% attained primary education or lower, and 34% achieved at least secondary education. More than half of the participants (58%) were registered as disabled.

**Table 3:** Social indicators among subjects (*n* = 100)

Social indicators	Frequency (%)	*P*-value (10 years)
**Education**		
Primary school or lower	7 (7.0)	0.332
Secondary school or higher	34 (34.0)	
Special school	45 (45.0)	
None	14 (14.0)	
**Registration as disabled**		
Yes	58 (58.0)	0.276
No	42 (42.0)	

### Relationship between seizure control and associated risk factors

The Wilcoxon signed-rank test was used to examine differences in seizure control from baseline through ten years of follow-up (Table 4). The results indicate that the post-test ranks from baseline and at 10 years did not show statistically significant differences compared to the pre-test ranks (*Z* = −0.577, *P* = 0.564). In addition, the relationship of individual variables to seizure freedom at baseline and at 10 years of follow-up was assessed using the chi-square test of independence. A Chi-square test of independence was performed to assess the relationship between seizure freedom and various demographic, social indicators, and treatment characteristics post-treatment, as shown in Tables 1 and 2, and 3. There was a significant relationship between seizure aetiology [χ²(5, 100) = 14.56, *P* = 0.006], subject behavioural issues [χ²(5, 100) = 26.87, *P* = 0.004], evidence of perinatal asphyxia [χ²(1, 100) = 3.409, *P* = 0.032], antiseizure medications [χ²(3, 100) = 9.694, *P* = 0.011] and seizure freedom at baseline. However, after 10 years of follow-up, antiseizure medication [χ²(3, 100) = 11.07, *P* = 0.006] was the only variable showing a significant relationship with seizure freedom, while all other variables were not statistically significant.

**Table 4:** Seizure control from onset of seizures to 10 years of follow-up

	Onset up to 10 years
*Z*	−0.577^b^
Asymp. Sig. (2-tailed)	0.564

b. Based on negative ranks

### Predictors of seizure freedom

The overall model was not statistically significant: chi-square (19) = 29.299, *P* = 0.061, Nagelkerke *R²*= 0.531. Individual predictors were also not statistically significant between seizure aetiology, behaviour, and antiseizure medication with seizure freedom, as shown in Table 5.

**Table 5:** Predictors of seizure freedom (Onset)

Variables		B	S.E.	df	*P*-value	OR	95% C.I.	
							Lower	Upper
**Seizure aetiology**	Structural			5	0.928			
	Genetic	−19.205	10696.875	1	0.999	0.000	0.000	
	Infection	−20.139	40192.970	1	1.000	0.000	0.000	
	Immune	40.774	45696.404	1	0.999	5105 × 10^14^	0.000	
	Unknown	−1.446	1.235	1	0.242	0.236	0.021	2.653
	Structural and genetic	−17.120	25955.316	1	0.999	0.000	0.000	
**Behaviour**	ASD			11	0.940			
	ADHD	−18.604	15621.232	1	0.999	0.000	0.000	
	Anxiety	22.626	40192.969	1	1.000	6704.71 × 10^7^	0.000	
	MDD	−18.841	21741.355	1	0.999	0.000	0.000	
	Mixed ADHD ASD	1.044	1.780	1	0.558	2.840	0.087	93.049
	Psychosis	22.626	40192.969	1	1.000	6704.71 × 10^6^	0.000	
	Aggression	0.825	2.359	1	0.726	2.282	0.022	232.260
	Adjustment disorder	−19.027	40192.970	1	1.000	0.000	0.000	
	Conversion disorder	−19.924	40192.970	1	1.000	0.000	0.000	
	OCD	−19.780	40192.970	1	1.000	0.000	0.000	
	None	−1.785	1.389	1	0.199	0.168	0.011	2.551
	Suicidal attempt	−20.139	40192.970	1	1.000	0.000	0.000	
	Perinatal asphyxia	−1.590	1.067	1	0.136	0.204	0.025	1.652
**Antiseizure medications**	Monotherapy			2	0.862			
	Dual therapy	−0.693	1.272	1	0.586	0.500	0.041	6.048
	Polytherapy	−0.334	1.208	1	0.782	0.716	0.067	7.643
	Constant	0.860	1.691	1	0.611	2.363		

## Discussion

This study provides longitudinal data from a local cohort of patients with childhood-onset epilepsy. As patients with self-limited epilepsy would likely default on long-term follow-up upon achieving remission, our final cohort leaned towards a higher proportion of drug-resistant cases. Most participants (72%) had intellectual or learning disabilities, while only a minority (12% each) had a family history of epilepsy or a perinatal event. Moreover, structural causes accounted for half of the aetiologies. Clinically, seizure semiology remained consistent over 10 years, with only 12% achieving seizure freedom, and no statistically significant difference in seizure control over 10 years. Pre-treatment EEGs were abnormal in over half of participants, most commonly showing focal epileptiform discharges.

### Sociodemographic factors

We recruited an equal number of male and female participants to minimise gender bias. Focal seizures were most common in our cohort, consistent with other studies reporting 45.0% to 56.5% prevalence [12–15]. This predominance is closely linked to the underlying aetiology, with many cases attributable to localized structural causes, including hypoxic-ischemic injuries or focal cortical malformations [13, 14] as demonstrated in this cohort. Importantly, unlike many childhood epilepsies that tend to remit, chronic epilepsy persisting into adulthood is predominantly focal in nature, reflecting the enduring impact of structural insults. Genetic causes were the second most common aetiology, with genetic generalized epilepsy (GGE) occurring in this age group [13–16]. A local study by Lim et al. also suggests a strong relationship between GGE and a positive family history [17, 18]. However, focal epilepsy can also be genetic, such as mutations in the leucine-rich glioma-inactivated 1 (LGI1) gene, which cause autosomal dominant focal epilepsy [19, 20]. Our cohort had fewer cases of unknown aetiology compared with most studies [13–16], likely due to advanced imaging and genetic testing at our tertiary centre. However, cost is still a limiting factor in pursuing these forms of investigations. In contrast, other multicentre studies may include hospitals with limited imaging resources and restricted access to genetic testing, particularly in earlier studies.

In our cohort, 72% of participants had an intellectual or learning disability, and 32% had behavioural issues, including ASD, ADHD, MDD, and mood disorders. The large proportion of mental disabilities in childhood epilepsy is similar to other published data, although data from newer studies remain limited [14, 15]. Typically, attention, concentration, memory, and executive function are the cognitive domains primarily affected [21]. These impairments are multifactorial, not solely attributable to structural or brain malformations. Important factors to consider include concurrent comorbidities, ASM side effects, dyslexia or other specific learning disabilities, and anxiety or depression related to stigma [21, 22]. Moreover, psychiatric conditions such as autism and mood disorders frequently coexist, with factors such as the age of epilepsy onset and a history of febrile seizures identified as some of the important risk factors [23, 24].

### Clinical characteristics

EEG is a fundamental tool in the diagnosis of epilepsy or an epilepsy syndrome. However, the sensitivity of a single routine EEG is limited, with studies indicating that approximately 50% of EEGs performed in patients with epilepsy were normal. This is likely due to the transient nature of epileptiform discharges, which might go undetected because of the short duration typically used in a routine EEG. Additionally, seizures originating from deep brain structures, such as mesial temporal lobe epilepsy, may not produce detectable scalp EEG abnormalities [25, 26]. Moreover, factors including the timing of the EEG, the patient’s state (e.g., wakefulness or sleep), and the number of recordings significantly affect detection rates. Extended EEG monitoring, sleep-deprived EEG, or repeated EEGs increase the diagnostic yield; however, a proportion of patients continue to have normal EEG findings despite clear clinical evidence of epilepsy [27]. Thus, epilepsy remains primarily a clinical diagnosis, whereby a normal EEG does not exclude it.

Interictal EEG changes, particularly interictal epileptiform discharges (IEDs), are common across all age groups in epilepsy. However, IEDs are occasionally observed in people without seizure disorders [28]. There is a relative paucity of studies investigating EEG changes following treatment. In our cohort, focal IEDs demonstrated a higher rate of post-treatment normalization, reaching up to 48.5%, compared with a 25.0% normalization rate in generalized IEDs. It is hypothesized that certain ASMs, particularly CBZ, can induce generalized IEDs [29].

Among the ASMs, VPA and CBZ were the most commonly used agents in childhood-onset epilepsy. VPA is particularly effective for generalized epilepsies, including childhood absence epilepsy, juvenile myoclonic epilepsy, and other idiopathic generalized epilepsies. Despite concerns regarding teratogenicity and metabolic side effects, valproate remains widely used in children, particularly boys, due to its superior efficacy in controlling multiple seizure types [30]. CBZ, in contrast, remains a first-line treatment for focal epilepsies in children, offering a favorable response profile and good tolerability in paediatric populations [31]. Routine HLA-B*15:02 screening significantly reduces the risk of Stevens-Johnson syndrome and toxic epidermal necrolysis, thereby improving its safety profile [32]. Additionally, both drugs have been included in the World Health Organization Model List of Essential Medicines for Children due to their accessibility and clinical utility across various resource settings [33]. While newer ASMs offer better side-effect profiles in some cases, VPA and CBZ remain widely used due to their effectiveness, availability, cost-effectiveness, and clinician familiarity.

### Care recommendations

Since our cohort excluded self-limiting childhood epilepsies and other childhood epilepsies that do not persist beyond childhood, for paediatric patients with seizure duration longer than 5 years approaching adolescence, a clear and well-established transition pathway from paediatric to adult neurology services is crucial. Currently, in most centres, patient care is transferred to adult neurology with a referral letter [34]. Yet, the transition should be individualized to address differing clinical and psychosocial needs. A multidisciplinary approach at a combined clinic, involving both paediatric and adult neurologists, a clinical psychologist, an epilepsy nurse, and a social worker or an epilepsy society representative, can address several issues [35, 36]. It allows each team to formally introduce themselves, thus building trust and alleviating the anxiety associated with the transfer of care. An epilepsy nurse is essential for assisting with follow-up, providing caregiver education and support, and serving as the first point of contact for patient concerns [37].

Additionally, clinical psychologists should be regularly introduced into epilepsy care. They are important to address the behavioural and learning disabilities that are prevalent among patients in most studies, including ours. Patients would benefit from cognitive behavioural therapy, practicing mindfulness, psychoeducation to manage their emotions and stress, and other self-management strategies [38]. Early screening for undiagnosed anxiety and depression would allow prompt intervention, which could have a positive clinical impact [39]. Patients who respond poorly to non-pharmacological modalities, should be referred for psychiatric assessment and pharmacotherapy when indicated.

In addition, structural neuroimaging should be performed whenever possible in childhood epilepsies, particularly when features are not characteristic of focal or generalized epilepsies [40]. A study by Samia et al. found that up to one-third of people with childhood epilepsy had detectable abnormalities on imaging. MRI is the preferred modality, although CT may be more accessible in certain settings [41, 42]. Identifying structural abnormalities is important, as it may reveal potentially reversible causes, including focal lesions amenable to surgical intervention.

Empowering non-governmental organizations or advocacy groups helps disseminate knowledge, provide financial and emotional support to patients and caregivers, and organize activities or awareness programs, which is crucial in epilepsy, where many individuals experience intellectual and behavioural disabilities. Research initiatives, such as the Managing Epilepsy Well network, are crucial in providing a focus for research direction and securing funding to support researchers [43]. In addition, peer support groups help to reduce perceived stigma among youths with epilepsy [44]. Technological advances have boosted the use of social media and online meeting platforms, facilitating interaction, information sharing, and emotional support [45].

In summary, we propose a 3-step transition plan to improve overall care. Phase 1 (Preparation, age 12–15) should include assessments by allied health personnel, such as a clinical psychologist, to screen for psychiatric issues. Furthermore, imaging is mandatory at this stage to exclude surgical lesions, particularly in drug-resistant epilepsy. Phase 2 (Joint assessment, age 16–18) involves joint consultations by peadiatric and adult neurologists to build trust, an epilepsy nurse to coordinate care and provide education, and an introduction to local NGOs. Phase 3 (Adult Assimilation, age > 18) executes the formal transfer to adult neurology to ensure long-term care continuity.

### Limitations

We recognize several limitations in this study. As this was a single-centre study, we cannot extrapolate the findings to the overall community. In addition, as a retrospective study, these findings cannot be generalized. A significant number of people with self-limited epilepsy who achieved seizure freedom within 10 years were not included in this cohort as they may have defaulted subsequent appointments; hence, the epilepsy remission rate was lower than 60% which was reported in most previous studies. Furthermore, of 117 screened patients, 17 were excluded due to missing key data, resulting in 100 participants, which is slightly below the target of 105 and potentially limiting the statistical power of some subgroup analyses. Moreover, due to the retrospective nature of the study design, we relied on documented medical records, which lacked the data required to confirm pre-existing cognitive impairment or changes in semiology. Consequently, this would have introduced a certain degree of selection bias. Moving forward, we aim to conduct a prospective, multicentre study involving other local and regional hospitals to address this research gap. Lastly, extending the study to at least a 20-year follow-up would provide valuable data, particularly on long-term outcomes.

## Conclusions

This study provides longitudinal data on childhood-onset epilepsy from a single tertiary centre, highlighting the complex interplay of clinical, neurophysiological, and psychosocial factors influencing long-term outcomes. Despite advancements in diagnosis and treatment, only a minority (12%) of patients achieved seizure freedom over 10 years, with no statistically significant change in seizure control over time. A substantial proportion had intellectual or learning disabilities (72%) and behavioural comorbidities, highlighting the broad neurodevelopmental impact of childhood-onset epilepsy beyond seizure control. These findings support the need for personalized, multidisciplinary management, including clear and structured transition plans from paediatric to adult neurology, alongside appropriate neuroimaging and integrated psychological and educational support.

## Data Availability

The datasets used and/or analysed during the current study are available from the corresponding author on reasonable request.

## Abbreviations

ADHD: Attention deficit hyperactivity disorder
ADL: Activities of daily living
ASD: Autism spectrum disorder
ASM: Antiseizure medication
ASMs: Anti-seizure medications
CBZ: Carbamazepine
CT: Computed tomography
DBS: Deep brain stimulation
EEG: Electroencephalography
GGE: Genetic generalised epilepsy
HIE: Hypoxic-ischemic encephalopathy
IEDs: Interictal epileptiform discharges
ILAE: International League Against Epilepsy
IQR: Interquartile range
LGI1: Leucine-rich glioma-inactivated 1
MCD: Malformation of cortical development
MDD: Major depressive disorder
MRI: Magnetic resonance imaging
OCD: Obsessive-compulsive disorder
PWE: People with epilepsy
RNS: Responsive neurostimulation
SD: Standard deviation
SPSS: Statistical Package for the Social Sciences
SUDEP: Sudden unexpected death in epilepsy
VNS: Vagus nerve stimulation
VPA: Sodium valproate
